# Two Cases of Reunion of Cut-off Fingers

**Published:** 1887-01

**Authors:** 


### Two Cases of Reunion of Cut-off Fingers.

In the *Russkaia Meditzina,* Dr. S. D. Ivanoff, of Briansk, furnishes details of two interesting cases occurring in healthy soldiers, one of whom had accidentlly cut away, by a stroke of an axe, the second phalanx of his right forefinger, while another had severed in the same way the second phalanx of his left thumb. In the former, the part hewn off was still united to the hand by means of a bridge of skin about two centimetres in breadth, the axe having passed through the first interphalangeal articulation, and chipped off a part of the base of the second phalanx. In the other case, the chopped off part was brought away in a glove; the weapon cut transversely a little above the interphalangeal articulation. In both of the cases the cut was clean, without any crushing of the parts. The first man was seen two hours after the accident; the second, three hours. The wounds were washed with a corrosive sublimate or carbolic solution, and the severed parts were accurately fixed by sutures in their normal situation, after which an iodoform dressing was applied. In both perfect union by the first intention ensued, with return of sensibility and (limited) mobility.

